# We All Gotta Go Sometime

**DOI:** 10.1371/journal.pbio.1000455

**Published:** 2010-08-17

**Authors:** Robin Meadows

**Affiliations:** Freelance Science Writer, Fairfield, California, United States of America

**Figure pbio-1000455-g001:**
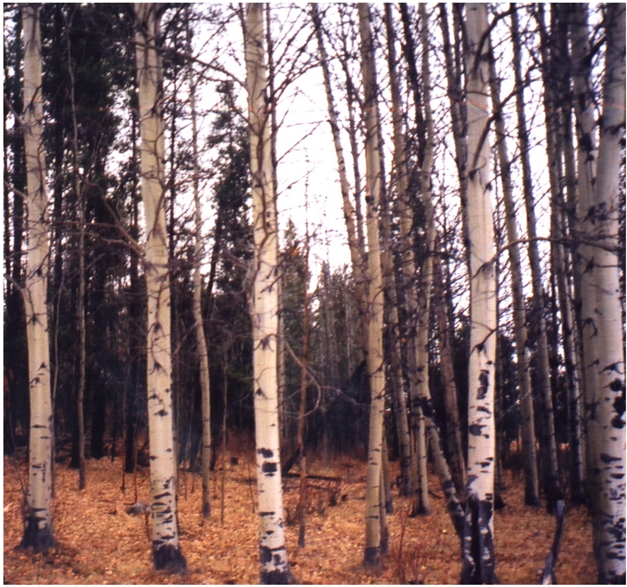
A forest stand of aspen. *Populus tremuloides*, like many other clonal plants, is capable of growing and reproducing throughout its life. Because clonal plants continually renew themselves, the question raised by these organisms is: do they age?

[Fig pbio-1000455-g001]Trembling aspens (*Populus tremuloides*) are among the world's most remarkable trees, forming vast clones that can live an improbably long time. These clones share a common root system, from which new trees arise, cover up to 43 hectares and persist up to a million years. The vigor of these ancient plants belies their years, leading to speculation that they defy aging or senescence. But demonstrating senescence or its absence in plants that live longer than we do is tricky. Now, in new research reported in this issue of *PLoS Biology*, Dilara Ally, Kermit Ritland, and Sarah Otto have overcome this obstacle partly by using declining fertility as a proxy for senescence.

Aging is thought to be a byproduct of natural selection acting most effectively earliest in life. Because species typically reproduce early in life, they can accumulate mutations that are deleterious late in life without jeopardizing their production of offspring. In contrast, clonal plants can also grow and reproduce sexually throughout their lives, raising the question of whether they senesce as they get older. Ally and colleagues thought they might for two reasons. First, longer-lived clones have had more cell divisions, increasing the accumulation of mutations in their “body” (somatic) cells. Second, because plant reproductive cells come from somatic cells, plants can pass mutations from their shoots and roots on to their offspring.

To investigate whether trembling aspens do senesce, Ally and colleagues asked if advanced age was linked to diminished fertility in clones. Age was estimated using a molecular clock, which was based on the amount of genetic diversity aspen clones had accumulated from the time since they were seeds. Aspen clones are either male or female, and fertility was based on the amount of viable pollen produced by male clones. To rule out the effects of environmental factors on fertility, the researchers accounted for influences such as disease, herbivory, and soil quality.

The aspen clones studied ranged from roughly 70 to 10,000 years old, and pollen analysis revealed a slow but steady loss of fertility with age. Fertility was cut by more than three-quarters in the oldest clone and, based on extrapolation, would likely have dwindled away entirely by 20,000 years. Thus, though it may take millennia, even plants that grow indefinitely will eventually succumb to old age.

Next the researchers investigated whether vegetative growth (asexual reproduction) comes at the expense of sexual reproduction in aspen clones. However, they found no evidence of tradeoffs between asexual and sexual reproduction. New shoots did not grow faster in aspen clones that were less fertile, older clones were not necessarily bigger than younger ones, and bigger clones were not less fertile than smaller ones. Alternative explanations for the age-related fertility drop include heritable epigenetic factors such as DNA methylation, which can affect reproductive functions from flowering to self-fertility.

These findings show that male trembling aspen clones are at risk of extinction as they lose fertility with age, because without sex they cannot disperse and start new clones. This work also raises a number of intriguing questions. Do male and female aspen clones lose fertility at different rates? Previous work suggests they might: sperm transmit more deleterious mutations than do eggs. And do other plants that form clones also lose fertility as they get older? Such astonishingly long-lived plants are found around the world, in habitats from land to sea, and include the Tasmanian tree *Lagarostrobos franklinii*, the Mediterranean sea grass *Posidonia oceanica*, and the western US fungus *Armillaria ostoyae*. The researchers' technique of using molecular clocks to estimate clone age opens up the possibility of answering these questions for a variety of ancient clonal plants in the wild.

**Ally D, Ritland K, Otto SP (2010) Aging in a Long-Lived Clonal Tree. doi:10.1371/journal.pbio.1000454**

